# Interaction Between Genetic Risk and Parental Feeding Practices in the Prediction of Overweight Across Adolescence

**DOI:** 10.1002/oby.70217

**Published:** 2026-05-11

**Authors:** Marthe de Roo, Tina Kretschmer, Clare Llewellyn, Bonamy R. Oliver, Catharina A. Hartman

**Affiliations:** ^1^ Faculty of Behavioral and Social Sciences, Department of Pedagogy and Educational Sciences University of Groningen Groningen the Netherlands; ^2^ Department of Psychology Friedrich‐Alexander University Erlangen‐Nuremberg Erlangen Germany; ^3^ Department of Behavioural Science and Health University College London London UK; ^4^ Department of Psychology and Human Development UCL Institute of Education, University College London London UK; ^5^ Interdisciplinary Center Psychopathology and Emotion Regulation (ICPE) University of Groningen, University Medical Center Groningen Groningen the Netherlands

**Keywords:** adolescence, gene–environment interaction, overweight development, parental feeding practices, polygenic score

## Abstract

**Objective:**

We examined whether genetic risk for higher BMI, parental feeding practices (restriction, pressure to eat, and monitoring), and their interactions were associated with overweight trajectories during adolescence.

**Methods:**

Data came from 5568 participants aged 10–18 in the Twins' Early Development Study who had at least one measurement of weight. Parental feeding practices were reported at age 10 using the Child Feeding Questionnaire. A polygenic score for BMI was used as a proxy for genetic risk. We identified overweight trajectories using latent class growth analysis, and associations with genetic risk, feeding practices, and their interactions were tested using multinomial logistic regression with trajectory as the outcome.

**Results:**

Three trajectories were identified: non‐overweight, persistent overweight, and adolescent‐onset overweight. Higher genetic risk and greater parental restriction and monitoring were associated with persistent overweight, whereas pressure to eat was associated with non‐overweight. Associations remained after adjustment for general parenting and additional risk factors. We found no evidence for gene–environment interaction.

**Conclusions:**

Genetic risk and parental feeding practices were independently associated with adolescent weight development. While this suggests feeding practices may be relevant targets for prevention, the findings likely represent complex bidirectional parent–child dynamics.

## Study Importance

- What is already known?
  ○ Parents, as gatekeepers of children's food access, may influence child weight development through feeding practices that shape food preferences and self‐regulation.
  ○ Evidence from observational studies is mixed, while randomized clinical trials in young children suggest that interventions targeting parental feeding practices are associated with short‐term child weight outcomes; however, few studies have examined these associations in adolescence, and it is unclear whether feeding practices interact with genetic risk to influence overweight development.
- What does this study add?
  ○ Parental feeding practices were associated with individual differences in developmental pathways of overweight during adolescence, over and above genetic risk for higher BMI, general parenting, and other risk factors.
  ○ No evidence was found that feeding practices moderated the associations between genetic risk and overweight trajectories.
- How might these results change the direction of research or the focus of clinical practice?
  ○ Future research should clarify whether, and at what developmental stage, parental feeding practices can effectively support youth at higher genetic risk for overweight.

## 1 Introduction

Overweight and obesity in children and adolescents pose a major public health challenge as prevalence rises and onset occurs at increasingly younger ages [1]. Globally, one in five individuals aged 5–19 years live with overweight, of whom 42% have obesity [2]. Children with obesity are five times more likely than their healthy‐weight peers to have obesity in adulthood and face elevated risks of serious health conditions [3, 4]. The limited long‐term success of treatment underscores the need to identify modifiable early‐life risk factors that can inform prevention interventions [5]. Both genetic and environmental factors contribute to overweight and obesity, with genetic influences becoming stronger during adolescence and into early adulthood [6]. Environmental factors have received considerable attention for their potential as prevention targets because they might modify genetic risk, a process referred to as gene–environment interaction (G × E) [7].

Although the family environment plays an important role in youth overweight development [8], most G × E research on weight‐related outcomes has focused on adults, with younger age groups receiving comparably less attention [9]. Parents, as gatekeepers of their children's food intake, are an important potential environmental source of variation in BMI trajectories during childhood and adolescence. Parental feeding practices have been hypothesized to influence children's eating habits by shaping food preferences and self‐regulation, which in turn affect weight outcomes [10]. Feeding practices include, for instance, monitoring, which refers to tracking a child's food intake, particularly of foods high in sugar or fat, to encourage healthy eating and prevent overconsumption. More coercive feeding practices include overt restriction, where parents impose strict limits on food access and intake, and pressure to eat, where parents insist or demand that their child eats more or finishes their plate, even when not hungry [10].

Observational studies on parental feeding practices and child weight have yielded mixed results. Associations between monitoring and child weight, for example, seem to depend on the intensity of monitoring [10]. Whereas moderate monitoring might encourage healthier food choices, excessive monitoring can backfire and lead to counterproductive eating habits [11]. Likewise, restriction, often driven by parents' good intentions to promote healthy eating or control weight, has been associated with less desirable eating behaviors. Limiting access to certain foods can heighten a child's desire for them and may increase the tendency to overeat [12]. However, observational evidence linking parental restriction to child weight outcomes remains inconsistent [10, 13]. Pressure to eat has also been examined in relation to child weight, with similarly inconsistent findings [10, 13]. Parents who pressure their child to eat more might see short‐term increases in intake, but repeated pressure can lead to food aversions over time, particularly toward healthy foods [14].

The timing and directionality of associations between parental feeding practices and child weight also remain unclear. For example, parents of children with a higher weight may increase monitoring or restriction in response to concerns about their child's weight [15]. Thus, feeding practices may reflect appropriate parental responses to child weight rather than unidirectional influences [16]. Relatively few longitudinal studies have explicitly modeled associations in both directions, leaving important gaps in our understanding of how parental feeding practices and child weight influence each other over time [13]. Extending these observational findings, well‐designed randomized clinical trials (RCTs) in infancy and early childhood have provided evidence that changing parental feeding can influence children's eating behavior and early growth, although effects on weight outcomes tend to be modest and do not persist beyond the preschool years [17, 18, 19].

It is possible that genetic predisposition for BMI interacts with feeding practices in shaping weight development. For example, children at higher genetic risk for overweight may benefit more from parental monitoring of food intake, as they tend to have lower satiety responsiveness and are more likely to overeat [20]. While previous G × E research on child and adolescent weight outcomes has primarily focused on distal environmental factors, such as family socioeconomic status [21, 22] and built neighborhood conditions [23], twin‐based findings also indicate that genetic influence on BMI is stronger among children living in more obesogenic home environments, suggesting that family contexts can modify genetic risk [9]. However, interactions between genetic risk and more proximal factors, such as parenting, remain poorly understood [24].

What is more, the interplay between genes and feeding practices is even more complex than G × E because feeding practices are not only influenced by parents' convictions and own eating‐related behaviors but also by child characteristics, including children's genetic predispositions. These predispositions shape child behaviors and traits that can, in turn, influence parenting through a mechanism known as evocative gene–environment correlation. Indeed, there is evidence that children with a genetic predisposition for higher BMI evoke greater parental restriction, whereas those with a lower genetic risk elicit parents to pressure them to eat [25].

In the current study, using longitudinal data spanning ages 10–18 years, we tested whether parental feeding practices moderated the association between genetic risk for higher BMI and overweight development during adolescence, modeled as latent class growth trajectories. While group‐based trajectory modeling is widely used to identify distinct weight trajectories [26], few studies have explored associations with G × E, particularly in adolescence [21]. Genetic risk was measured using a polygenic score for BMI. Because genetic risk and feeding practices may co‐occur due to family‐level characteristics (e.g., socioeconomic disadvantage, parental overweight), we accounted for these potential sources of confounding when estimating G × E [27].

To examine whether feeding practices were uniquely associated with overweight development, we also assessed whether associations were independent of general parenting.

Based on previous research, we expected to identify three distinct overweight trajectories: a group without overweight, a group with persistent overweight, and a group that develops overweight during adolescence [26]. We hypothesized that higher genetic predisposition for overweight would increase the likelihood for an individual to follow a persistent overweight trajectory, relative to the non‐overweight trajectory. Building on theory [10], we also hypothesized that greater parental monitoring would attenuate the effect of genetic predisposition on overweight trajectories. Finally, we examined the moderating roles of parental restriction and pressure to eat, but in an exploratory manner, as prior research has reported mixed findings for these practices, providing no clear basis for specific hypotheses regarding their interactions with genetic predisposition.

## 2 Methods

Methods and hypotheses were preregistered on the Open Science Framework prior to accessing the data (https://osf.io/jktec).

### 2.1 Participants

We used data from the Twins' Early Development Study (TEDS), a longitudinal study of twin pairs born in England and Wales between 1994 and 1996, identified through birth records [28]. The first wave of data collection occurred at 18 months, with 13,759 families (50.0% female) participating. Twins have since been assessed at least biennially through young adulthood, with the most recent wave completed at age 26. TEDS families are broadly representative of the population in England and Wales in terms of ethnicity and socioeconomic factors. Written consent was obtained from all participating parents and twins, and the study was approved by the King's College London ethics committee.

This study included data from six assessment waves, when twins were 9, 10, 12, 14, 16, and 18 years old. We excluded participants due to serious medical conditions, extreme perinatal complications, missing essential background data, or unknown zygosity or sex. After exclusions, 6973 unrelated genotyped individuals were available for analysis, with one twin randomly selected from each pair so that only one child per family was included.

### 2.2 Measures

#### 2.2.1 Overweight

Height and weight were parent‐reported at age 10 using standardized instructions. Correlations between researcher‐measured and parent‐reported height and weight in a subsample (*n* = 288) were 0.90 and 0.83, respectively [29]. At ages 12, 14, 16, and 18, height and weight were self‐reported. BMI was calculated as weight divided by height squared (kg/m^2^) and dichotomized (*0 = non‐overweight* and *1 = overweight or obesity*) using age‐ and sex‐specific cutoffs from the International Obesity Task Force (IOTF) [30].

#### 2.2.2 Polygenic Score for BMI

Details on DNA extraction, genotyping, and quality control are documented elsewhere [25]. We used a polygenic score for BMI based on summary statistics from a GWAS of ~700,000 adults of European ancestry [31]. Although this score may not capture all genetic variants specific to adolescent overweight development, adult‐derived polygenic scores have been shown to predict adolescent BMI with similar accuracy to BMI in adults [32]. The score was calculated using LDpred2‐auto with default parameters [33]. Only HapMap3+ variants were included, and the target sample served as the linkage disequilibrium reference panel. All available SNPs were included (*n* = 868,323), and the polygenic score was standardized (mean = 0, standard deviation [SD] = 1).

#### 2.2.3 Parental Feeding Practices

The Child Feeding Questionnaire (CFQ) was used to assess parental feeding practices and was completed by parents when their twins were 10 years old [34]. Three scales were used: Restriction (six items; e.g., “I have to make sure that my child does not eat too many sweet things”; *α* = 0.79), Pressure to eat (four items; e.g., “My child should always eat all of the food on his/her plate”; *α* = 0.60), and Monitoring (three items; e.g., “How much do you keep track of the sweet things your child eats?”; *α* = 0.87). Items were rated on a 5‐point Likert scale and mean composite scores were calculated for each subscale, with higher scores indicating greater monitoring, restriction, or pressure to eat.

#### 2.2.4 General Parenting Measures

Harsh parental discipline was assessed at age 9 using two parent‐reported items on negative disciplinary practices (e.g., “I tell him or her off or shout at him or her”), rated 0–4, with higher scores indicating more harsh discipline. Positive parental feelings were measured using three items (e.g., “I feel happy about my relationship with my child”), rated 0–2, with higher scores indicating more positive feelings.

#### 2.2.5 Covariates

Covariates included sex, age at baseline, socioeconomic status, parental BMI, and pubertal development. Socioeconomic status was assessed at first contact based on maternal and paternal educational levels, maternal and paternal employment levels, and maternal age at first birth. The overall score was calculated as the mean of the five standardized items. Pubertal development was assessed at age 12 using a self‐report measure of pubertal status, including three common and two sex‐specific items, with higher scores indicating more mature physical development [35]. Parental BMI (kg/m^2^) was assessed using self‐reported height and weight when twins were age 10 (96.3% mothers). Models including the polygenic score were also adjusted for 10 principal components and chip type.

### 2.3 Analytic Strategy

We first estimated a single‐class latent growth model of overweight status using Mplus version 8.6 (Muthén & Muthén). To account for unequal spacing between assessment waves, we defined the time metric as average years since baseline: 0.0 for age 10, 1.4 for age 12, 4.1 for age 14, 6.4 for age 16, and 8.7 for age 18. We estimated an intercept‐only model, a linear growth model, and a model including a quadratic term. Models were fitted using full information maximum likelihood to handle missing data. Thus, the latent class growth models included all participants with at least one data point for overweight status (*n* = 5568). We then determined the optimal number of latent classes by estimating latent class growth models with an increasing number of classes. Model fit was evaluated using BIC (lower values indicate better fit), LMR‐LRT, BLRT, average classification probability (values closer to one indicate better classification), entropy (values closer to one indicate better fit), and theoretical expectations and interpretability. We examined potential sex differences by estimating the selected model separately for boys and girls.

Prior to the main analyses, we inspected scatterplots of each parental feeding practice and overweight status at age 10 and compared mean feeding practice scores across trajectory classes to confirm that associations between feeding practices and overweight were approximately linear. We then examined associations between the polygenic score for BMI, parental feeding practices, and the overweight trajectories using multinomial logistic regression. Predictors of class membership were included as auxiliary variables using the three‐step approach with adjustment for classification errors [36].

We entered the polygenic score for BMI as a predictor of the trajectories in the first step to test for main genetic effects (Model 1). In the next step, we added each feeding practice (i.e., restriction, pressure to eat, and monitoring) in a separate model as an additional predictor alongside the polygenic score for BMI (Models 2a–4a). In the final step, we added interaction terms between the polygenic score for BMI and the respective feeding practice to test for G × E.

To reduce potential bias in G × E estimates arising from family‐level characteristics associated with both genetic risk and parental feeding practices, we additionally included interaction terms between each feeding practice and socioeconomic status and between each feeding practice and parental BMI (Models 2b–4b). Attenuation of G × E estimates after inclusion of these terms would suggest that the initial interaction effects were at least partly influenced by such confounding [27].

As a negative validation check, we added general parenting measures (harsh discipline and positive parental feelings) to models where main associations between feeding practices and overweight trajectories were significant. In models with significant G × E terms, we also included interactions between the general parenting measures and the polygenic score for BMI.

Continuous predictors were mean‐centered prior to the G × E analyses to reduce multicollinearity. Missing data on environmental variables and covariates were addressed using multiple imputation (*m* = 20, 50 iterations per imputation; SPSS's Fully Conditional Specification Method). We created composite scales prior to imputation. Interaction terms were incorporated into the imputation model. Analyses were conducted in the imputed datasets using Mplus, and pooled estimates are reported.

## 3 Results

Table 1 presents descriptive statistics of the analytic sample (*n* = 5568). The polygenic score for BMI explained up to 12.6% of BMI variance, adjusted for sex, age, and 10 principal components (Figure S1). The full correlation matrix is provided in Table S1 and visualized in Figure S2. At age 10, overweight status correlated modestly with feeding practices (Restriction *r*_*pb*_ = 0.16, Pressure to eat *r*_*pb*_ = −0.17, Monitoring *r*_*pb*_ = 0.03). The BMI polygenic score showed small correlations with feeding practices (Restriction *r* = 0.10, Pressure to eat *r* = −0.08, Monitoring *r* = 0.02), suggesting some degree of gene–environment correlation. Scatterplots and mean feeding practice scores across trajectory classes showed approximately linear associations between feeding practices and overweight.

**TABLE 1:** Descriptive statistics of the analytic sample (*n* = 5568) before imputation.

Variable	Mean ± SD or percentage	*n* total	%‐missing
Overweight (age 10)^a^	13.0% overweight	4314	22.5%
Overweight (age 12)^b^	12.5% overweight	3915	29.7%
Overweight (age 14)^c^	10.1% overweight	2162	61.2%
Overweight (age 16)^d^	12.4% overweight	1708	69.3%
Overweight (age 18)^e^	16.8% overweight	1223	78.0%
Polygenic score for BMI	0 ± 1	5568	0%
Restriction	3.15 ± 1.03	4337	22.1%
Pressure to eat	2.46 ± 0.92	4395	21.1%
Monitoring	2.98 ± 0.88	4416	20.7%
*Covariates*			
Sex	46.8% male	5568	0%
Age (baseline)	9.91 ± 0.86	4443	20.2%
Socioeconomic status	0 ± 1	5283	5.1%
Parental BMI	25.2 ± 4.55	4024	27.7%
Harsh discipline	1.55 ± 0.74	2416	56.6%
Positive parental feelings	5.56 ± 0.77	2418	56.6%
Puberty scale	1.70 ± 0.56	4505	19.1%

*Note*: Descriptive statistics are based on participants with at least one measurement of weight (*n* = 5568).

^a^ BMI cutoff for overweight in boys was 19.84 kg/m^2^; cutoff for girls was 19.86 kg/m^2^.

^b^ BMI cutoff for overweight in boys was 20.89 kg/m^2^; cutoff for girls was 21.20 kg/m^2^.

^c^ BMI cutoff for overweight in boys was 22.62 kg/m^2^; cutoff for girls was 23.34 kg/m^2^.

^d^ BMI cutoff for overweight in boys was 24.19 kg/m^2^; cutoff for girls was 24.54 kg/m^2^.

^e^ BMI cutoff for overweight was 25 kg/m^2^ for both boys and girls.

### 3.1 Developmental Trajectories of Overweight Status

**FIGURE 1:**
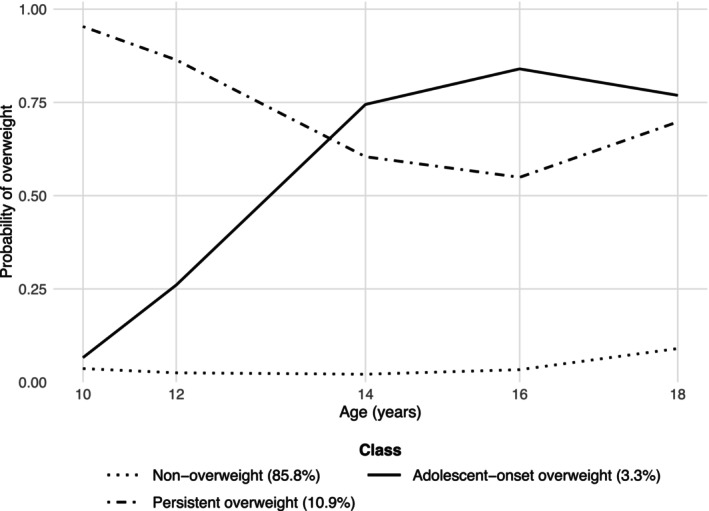
Trajectories of overweight status from the best‐fitting three‐class model (*n* = 5568). Reported class sizes are based on posterior probabilities.

We fitted latent class growth models to identify developmental trajectories of overweight status. A quadratic growth term improved model fit compared to the linear model, as indicated by a lower BIC and a significant quadratic term in the single‐class solution. Based on the minimized BIC, high entropy (0.84), and theoretical expectations, a three‐class model provided the best fit. This solution fit significantly better than the two‐class model (LMR‐LRT = 36.44, *p* < 0.001; BLRT = 37.50, *p* < 0.001). Adding a fourth class did not improve fit according to BIC, entropy, or LMR‐LRT. The three‐class model identified a large group without overweight (85.6%, *n* = 4779; posterior probability = 0.96), a group with persistent overweight (10.8%, *n* = 604; posterior probability = 0.85), and a smaller group with adolescent‐onset overweight (3.3%, *n* = 182; posterior probability = 0.71) (Figure 1). The single‐class model suggested minor sex differences, but three‐class trajectories estimated separately by sex were similar, particularly for the classes with non‐overweight and persistent overweight, justifying a combined analysis. Full model fit indices and parameter estimates are provided in Tables S2–S4.

### 3.2 Polygenic Score for BMI and Parental Feeding Practices as Predictors of Overweight Trajectories

Each SD increase in genetic risk was associated with 2.14 times higher odds (95% confidence interval [CI] [1.86, 2.46]) of belonging to the group with persistent overweight compared to the group without overweight (Table 2, Model 1). Genetic risk was not associated with a higher likelihood of belonging to the adolescent‐onset group compared to either the group without overweight or the group with persistent overweight.

**TABLE 2:** Key results of multinomial logistic regression models predicting class membership from the polygenic score for BMI (Model 1), parental feeding practices (Models 2a–4a), and their interactions (Models 2b–4b).

Model	Predictor	Adolescent‐onset overweight versus non‐overweight (ref)		Persistent overweight versus non‐overweight (ref)		Persistent overweight versus adolescent‐onset overweight (ref)	
		OR (SE)	95% CI	OR (SE)	95% CI	OR (SE)	95% CI
1.	Polygenic score for BMI	3.01 (1.82)	[0.93, 9.81]	**2.14 (0.15)** *	**[1.86, 2.46]**	0.71 (0.43)	[0.22, 2.32]
2a.	Restriction	2.02 (1.25)	[0.60, 6.78]	**1.62 (0.13)** *	**[1.38, 1.90]**	0.80 (0.52)	[0.23, 2.85]
2b.	PGS_*BMI*_ × Restriction	1.30 (1.12)	[0.24, 7.06]	1.04 (0.10)	[0.85, 1.27]	0.80 (0.72)	[0.14, 4.64]
3a.	Pressure to eat	0.97 (0.47)	[0.37, 2.49]	**0.49 (0.04)** *	**[0.42, 0.58]**	0.51 (0.24)	[0.20, 1.29]
3b.	PGS_*BMI*_ × Pressure to eat	0.93 (0.58)	[0.27, 3.14]	0.83 (0.08)	[0.68, 1.01]	0.90 (0.57)	[0.26, 3.14]
4a.	Monitoring	0.94 (0.38)	[0.43, 2.06]	**1.17 (0.09)** *	**[1.00, 1.36]**	1.24 (0.51)	[0.55, 2.78]
4b.	PGS_*BMI*_ × Monitoring	0.90 (0.55)	[0.27, 2.97]	1.17 (0.10)	[0.99, 1.40]	1.31 (0.81)	[0.39, 4.43]

*Note*: ORs reflect the likelihood of belonging to each higher‐risk trajectory relative to the reference class (ref). Analyses were based on *n* = 5568 participants. All models were adjusted for sex, age, socioeconomic status, parental BMI, and puberty. Models including the polygenic score for BMI were adjusted for the first 10 principal components and chip type. Full model results are presented in Table S11.

Abbreviations: OR, odds ratio; PGS_*BMI*_, polygenic score for BMI; SE, standard error.

* *p* < 0.05. Bold values indicate statistically significant associations.

All parental feeding practices distinguished individuals in the non‐overweight and persistent overweight groups after adjusting for genetic risk. Specifically, greater use of restriction and monitoring was associated with 62% higher odds (odds ratio [OR] = 1.62, 95% CI [1.38, 1.90]) and 17% higher odds (OR = 1.17, 95% CI [1.00, 1.36]), respectively, of belonging to the group with persistent overweight, whereas more pressure to eat was associated with 49% lower odds (OR = 0.49, 95% CI [0.42, 0.58]). Feeding practices did not differentiate the adolescent‐onset group from the other trajectories. Associations between genetic risk and class membership remained similar in magnitude after adding the parental feeding practices, indicating independent contributions of each (Table S5).

After adjusting for general parenting measures, the associations between parental feeding practices and class membership were largely unchanged (Tables S6–S8). The general parenting measures did not predict class membership in any model.

### 3.3 Gene–Environment Interactions

We found no evidence that parental feeding practices moderated the association between genetic risk and class membership (Table 2, Models 2b–4b). Adjusting for potential confounding by including interactions between feeding practices and socioeconomic status and parental BMI did not alter the G × E estimates; unadjusted G × E models yielded similar results (Tables S9–S11). Given the absence of G × E, we did not estimate models including interactions between general parenting measures and the polygenic score for BMI as a negative validation check. In all models, the confidence intervals for comparisons involving the adolescent‐onset group were wide, reflecting greater uncertainty in these estimates.

## 4 Discussion

In this longitudinal study spanning 8 years, we identified three overweight trajectories: non‐overweight, adolescent‐onset, and persistent overweight, consistent with previous research [26]. As expected, the polygenic score for BMI distinguished the persistent overweight from the non‐overweight trajectory, such that higher genetic risk was associated with an increased likelihood of following a developmental pattern of persistent overweight through late childhood and adolescence. The polygenic score did not differentiate the adolescent‐onset trajectory from the other trajectories. It is possible that adolescent‐onset overweight reflects a developmental pathway in which environmental factors play a comparatively larger role, consistent with prior research indicating that children at higher genetic risk typically gain weight earlier, even before adolescence [37]. However, the adolescent‐onset group was small (3.3% of the total sample) and showed relatively low classification precision (posterior probability = 0.71), which may have reduced statistical power and increased uncertainty in estimates involving this group.

Parental feeding practices distinguished the group with persistent overweight from the group without overweight over and above genetic risk and other established risk factors for overweight, including socioeconomic status and parental BMI. Children were more likely to follow a developmental pattern of persistent overweight when parents reported greater use of restriction and monitoring, whereas pressure to eat was associated with non‐overweight. Associations remained after adjusting for general parenting measures, suggesting that feeding practices are uniquely associated with overweight development, distinct from broader parenting behaviors. These findings are consistent with research suggesting that parental attempts to manage their children's eating, for example through restriction, may be associated with counterproductive eating behaviors over time, including increased intake of restricted foods [11, 12, 14]. At the same time, it is also plausible that child characteristics shape parental feeding practices.

Specifically, feeding practices may reflect parental responses to child traits, such as early overweight status or appetite, that are themselves genetically influenced. Overweight status at baseline correlated with feeding practices, supporting the possibility of such evocative gene–environment correlation. Given that weight status was already established for many in the group with persistent overweight when feeding practices were measured, these practices may reflect adaptations to the child's existing weight rather than behaviors that preceded or shaped overweight development. Adjusting for earlier child weight would not necessarily clarify this distinction, as prior weight is likely both a determinant and an outcome of parental feeding practices. Stronger causal inference would require repeated longitudinal assessments of both feeding and offspring weight. Feeding practices were assessed at two earlier ages in TEDS, but those measures were less extensive and not directly comparable to the CFQ assessment used at age 10. RCTs in infancy and early childhood have partly addressed this gap by demonstrating that feeding practices can be modified through intervention, with some evidence that these interventions are associated with changes in children's eating behavior and short‐term weight outcomes [17, 18, 19]. To our knowledge, interventions specifically targeting parental feeding practices have not yet been tested in adolescence, leaving open the question of whether modifying feeding practices remains effective once children gain greater autonomy over eating.

We found no evidence that parental feeding practices modified genetic risk. As parental involvement in their children's eating likely declines across development [10], such interactions may be more relevant earlier in childhood. At the same time, using polygenic scores with greater biological specificity may reveal interaction patterns that genome‐wide scores, such as the one applied in this study, obscure [38]. For example, polygenic scores capturing pathways involved in metabolism or reward‐related mechanisms could provide more interpretable insights [39].

While not part of our initial hypotheses, we observed that higher parental BMI was associated with an increased likelihood of following a persistent or adolescent‐onset trajectory compared to the non‐overweight trajectory, even after adjusting for child genetic risk. One explanation is that parental BMI partially captured unmeasured genetic risk, as polygenic scores currently account for only a fraction of the total genetic influence on BMI [40]. Another possibility involves genetic nurture, whereby parents' genetically influenced traits, reflected in their BMI and expressed through behaviors such as feeding practices, shape the family environment in ways that promote weight gain in offspring. Some evidence for genetic nurture comes from recent studies showing that maternal BMI‐related genetic variants not transmitted to the child are associated with offspring BMI in childhood and adolescence [41, 42]. However, another study focusing on childhood overweight found no such effect [43].

Although the design of our study does not allow for causal conclusions or direct recommendations for interventions, our findings tentatively suggest that parental feeding practices, as modifiable factors, warrant further investigation as potential targets for preventive strategies. Simulation work has suggested that changing feeding practices could reduce disparities in BMI associated with genetic risk [44]. While most prior studies have focused on early childhood, our findings extend this work by examining these associations in adolescence and by integrating genetic risk, highlighting the need to understand how the relevance of feeding practices may change across development.

The longitudinal data, inclusion of genetic information, and rigorous testing of gene–environment interaction, were important strengths of this study. Nonetheless, some limitations should be acknowledged. First, height and weight were self‐reported from age 12 onwards. In adolescents, self‐reported height and weight show moderate to high agreement with objectively measured values, but BMI is typically slightly underestimated due to underreporting of weight and overreporting of height [45]. This may have resulted in an underestimation of overweight prevalence and might have attenuated associations. Second, feeding practices and parental BMI were self‐reported by one parent, most often the mother, so these measures primarily reflect maternal behaviors. Third, the pressure to eat scale showed relatively low internal consistency, which may have introduced measurement error in models involving this construct. Fourth, 1419 participants had only a single data point for overweight status. These individuals were retained in the latent class growth model to maintain statistical power, but their limited data may have reduced the precision of the trajectory estimates. Fifth, although parents likely use a combination of feeding strategies, we analyzed each practice separately due to modest intercorrelations and to limit model complexity. Sixth, some feeding practices, particularly pressure to eat, may also be associated with underweight. Only a small number of participants were in the underweight category at two or more assessments (*n* = 68), and excluding them did not alter the conclusions (Table S12). Finally, associations between feeding practices and child weight may differ across cultural and ancestral contexts, and findings should be replicated in more diverse populations.

## 5 Conclusion

Our study demonstrated the likely independent contributions of child genetic risk and parental feeding practices to overweight development across adolescence, over and above general parenting and other risk factors for overweight, with no evidence of gene–environment interaction. These associations may reflect both parental influences on offspring weight and parental responses to children's genetically influenced traits, highlighting the complex bidirectional relationships between parent and child. Future research should examine whether the moderating role of parental feeding varies across development, particularly during early childhood when parental involvement in children's eating is greatest.

## Funding

T.K. is funded by the European Research Council Grant under the Horizon Europe program (grant agreement 101087395; title: “PAR2: A new science of parenting”). C.A.H. acknowledges the funding of TIMESPAN by the European Union's Horizon 2020 Research and Innovation program under grant agreement 965381. Funding was also provided by Medical Research Council, MR/V012878/1; National Institutes of Health, AG046938.

## Conflicts of Interest

The authors declare no conflicts of interest.

## Supporting information


**Figure S1:** Association of the polygenic score for BMI with BMI in TEDS (*N* = 6973).
**Figure S2:** Heat map of correlations.
**Table S1:** Pairwise correlations between variables in the study.
**Table S2:** Model fit indices for latent class growth analyses of overweight status. Growth is defined by intercepts and linear and quadratic slopes.
**Table S3:** Classification probabilities for the three‐class model. Average latent class probabilities for most likely latent class membership (row) by latent class (column).
**Table S4:** Growth parameters for the three‐class model.
**Table S5:** Full results of multinomial logistic regression models predicting class membership from the polygenic score for BMI (Model 1), parental feeding practices (Models 2a–4a), and their interactions (Models 2b–4b) (corresponding to Table 2).
**Table S6:** Negative validation check: Restriction.
**Table S7:** Negative validation check: Pressure to eat.
**Table S8:** Negative validation check: Monitoring.
**Table S9:** Unadjusted G × E model: Polygenic score for BMI × Restriction.
**Table S10:** Unadjusted G × E model: Polygenic score for BMI × Pressure to eat.
**Table S11:** Unadjusted G × E model: Polygenic score for BMI × Monitoring.
**Table S12:** Follow‐up analyses excluding participants with underweight: Main effects of the polygenic score for BMI (Model 1), parental feeding practices (Models 2a–4a), and polygenic score for BMI × feeding practices (Models 2b–4b) in the prediction of class membership.

## Data Availability

Data used for this submission may be made available on request to the Twins Early Development Study (TEDS), through its data access mechanism (https://www.teds.ac.uk/researchers/teds‐data‐access‐policy/). We will then consider requests for sharing data for appropriate research purposes.

## References

[1] NCD Risk Factor Collaboration (NCD‐RisC), “Worldwide Trends in Underweight and Obesity From 1990 to 2022: A Pooled Analysis of 3663 Population‐Representative Studies With 222 Million Children, Adolescents, and Adults,” Lancet 403, no. 10431 (2024): 1027–1050, 10.1016/S0140-6736(23)02750-2. 38432237 PMC7615769

[2] United Nations Children's Fund (UNICEF) , Feeding Profit: How Food Environments Are Failing Children – 2025 Child Nutrition Report (UNICEF, 2025).

[3] M. Simmonds , A. Llewellyn , C. G. Owen , and N. Woolacott , “Predicting Adult Obesity From Childhood Obesity: A Systematic Review and Meta‐Analysis,” Obesity Reviews 17, no. 2 (2016): 95–107, 10.1111/obr.12334. 26696565

[4] A. Sommer and G. Twig , “The Impact of Childhood and Adolescent Obesity on Cardiovascular Risk in Adulthood: A Systematic Review,” Current Diabetes Reports 18, no. 10 (2018): 91, 10.1007/s11892-018-1062-9. 30167798

[5] L. J. Ells , K. Rees , T. Brown , et al., “Interventions for Treating Children and Adolescents With Overweight and Obesity: An Overview of Cochrane Reviews,” International Journal of Obesity (Lond) 42, no. 11 (2018): 1823–1833, 10.1038/s41366-018-0230-y. 30301964

[6] K. Silventoinen , A. Jelenkovic , R. Sund , et al., “Genetic and Environmental Effects on Body Mass Index From Infancy to the Onset of Adulthood: An Individual‐Based Pooled Analysis of 45 Twin Cohorts Participating in the Collaborative Project of Development of Anthropometrical Measures in Twins (CODATwins) Study,” American Journal of Clinical Nutrition 104, no. 2 (2016): 371–379, 10.3945/ajcn.116.130252. 27413137 PMC4962159

[7] B. Domingue , S. Trejo , E. Armstrong‐Carter , and E. Tucker‐Drob , “Interactions Between Polygenic Scores and Environments: Methodological and Conceptual Challenges,” Sociological Sciences 7 (2020): 465–486, 10.15195/v7.a19. PMC9455807 36091972

[8] A. R. Kininmonth , A. D. Smith , C. H. Llewellyn , L. Dye , C. L. Lawton , and A. Fildes , “The Relationship Between the Home Environment and Child Adiposity: A Systematic Review,” International Journal of Behavioral Nutrition and Physical Activity 18, no. 1 (2021): 4, 10.1186/s12966-020-01073-9. 33407598 PMC7788808

[9] S. Schrempft , C. H. M. van Jaarsveld , A. Fisher , et al., “Variation in the Heritability of Child Body Mass Index by Obesogenic Home Environment,” JAMA Pediatrics 172, no. 12 (2018): 1153–1160, 10.1001/jamapediatrics.2018.1508. 30285028 PMC6396810

[10] A. E. Vaughn , D. S. Ward , J. O. Fisher , et al., “Fundamental Constructs in Food Parenting Practices: A Content Map to Guide Future Research,” Nutrition Reviews 74, no. 2 (2016): 98–117, 10.1093/nutrit/nuv061. 26724487 PMC4892304

[11] A. E. Mellin , D. Neumark‐Sztainer , M. Story , M. Ireland , and M. D. Resnick , “Unhealthy Behaviors and Psychosocial Difficulties Among Overweight Adolescents: The Potential Impact of Familial Factors,” Journal of Adolescent Health 31, no. 2 (2002): 145–153, 10.1016/S1054-139X(01)00396-2. 12127384

[12] P. DeCosta , P. Møller , M. B. Frøst , and A. Olsen , “Changing Children's Eating Behaviour—A Review of Experimental Research,” Appetite 113 (2017): 327–357, 10.1016/j.appet.2017.03.004. 28286164

[13] D. Beckers , L. T. Karssen , J. M. Vink , W. J. Burk , and J. K. Larsen , “Food Parenting Practices and Children's Weight Outcomes: A Systematic Review of Prospective Studies,” Appetite 158 (2021): 105010, 10.1016/j.appet.2020.105010. 33075443

[14] A. T. Galloway , L. M. Fiorito , L. A. Francis , and L. L. Birch , “‘Finish Your Soup’: Counterproductive Effects of Pressuring Children to Eat on Intake and Affect,” Appetite 46, no. 3 (2006): 318–323, 10.1016/j.appet.2006.01.019. 16626838 PMC2604806

[15] I. P. Derks , H. Tiemeier , E. J. Sijbrands , et al., “Testing the Direction of Effects Between Child Body Composition and Restrictive Feeding Practices: Results From a Population‐Based Cohort,” American Journal of Clinical Nutrition 106, no. 3 (2017): 783–790, 10.3945/ajcn.117.156448. 28793987

[16] C. V. Farrow and J. Blissett , “Controlling Feeding Practices: Cause or Consequence of Early Child Weight?,” Pediatrics 121, no. 1 (2008): e164–e169, 10.1542/peds.2006-3437. 18166535

[17] A. Magarey , C. Mauch , K. Mallan , et al., “Child Dietary and Eating Behavior Outcomes up to 3.5 Years After an Early Feeding Intervention: The NOURISH RCT,” Obesity (Silver Spring) 24, no. 7 (2016): 1537–1545, 10.1002/oby.21498. 27193736

[18] J. S. Savage , E. E. Hohman , M. E. Marini , A. Shelly , I. M. Paul , and L. L. Birch , “INSIGHT Responsive Parenting Intervention and Infant Feeding Practices: Randomized Clinical Trial,” International Journal of Behavioral Nutrition and Physical Activity 15, no. 1 (2018): 64, 10.1186/s12966-018-0700-6. 29986721 PMC6038199

[19] I. M. Paul , J. S. Savage , S. Anzman‐Frasca , et al., “Effect of a Responsive Parenting Educational Intervention on Childhood Weight Outcomes at 3 Years of Age: The INSIGHT Randomized Clinical Trial,” JAMA 320, no. 5 (2018): 461–468, 10.1001/jama.2018.9432. 30088009 PMC6142990

[20] C. H. Llewellyn , M. Trzaskowski , C. H. M. van Jaarsveld , R. Plomin , and J. Wardle , “Satiety Mechanisms in Genetic Risk of Obesity,” JAMA Pediatrics 168, no. 4 (2014): 338–344, 10.1001/jamapediatrics.2013.4944. 24535189 PMC3981891

[21] M. de Roo , C. Hartman , R. Veenstra , et al., “Gene‐Environment Interplay in the Development of Overweight,” Journal of Adolescent Health 73, no. 3 (2023): 574–581, 10.1016/j.jadohealth.2023.04.028. 37318409

[22] J. A. Kerr , D. Dumuid , M. Downes , et al., “Socioeconomic Disadvantage and Polygenic Risk of Overweight in Early and Mid‐Life: A Longitudinal Population Cohort Study Spanning 12 Years,” Lancet Regional Health – Western Pacific 53 (2024): 101231, 10.1016/j.lanwpc.2024.101231. 39624156 PMC11609315

[23] M. de Roo , C. A. Hartman , A. Wagtendonk , H. W. Hoek , J. Lakerveld , and T. Kretschmer , “Interplay Between Genetic Risk and Built Neighborhood Conditions as Predictor of BMI Across the Transition Into Adulthood,” Obesity (Silver Spring) 33, no. 2 (2025): 385–394, 10.1002/oby.24213. 39828653 PMC11774011

[24] M. de Roo , C. A. Hartman , M. Wiertsema , and T. Kretschmer , “Gene‐Environment Interplay Explaining Individual Variation in BMI Outcomes: A Systematic Review and Meta‐Analysis of Studies Using Polygenic Indices,” International Journal of Obesity 50 (2026): 268–287, 10.1038/s41366-025-01957-5. 41318678

[25] S. Selzam , T. A. McAdams , J. R. I. Coleman , et al., “Evidence for Gene‐Environment Correlation in Child Feeding: Links Between Common Genetic Variation for BMI in Children and Parental Feeding Practices,” PLoS Genetics 14, no. 11 (2018): e1007757, 10.1371/journal.pgen.1007757. 30457987 PMC6245504

[26] V. De Rubeis , A. T. Andreacchi , I. Sharpe , L. E. Griffith , C. D. G. Keown‐Stoneman , and L. N. Anderson , “Group‐Based Trajectory Modeling of Body Mass Index and Body Size Over the Life Course: A Scoping Review,” Obesity Science and Practice 7, no. 1 (2021): 100–128, 10.1002/osp4.456.

[27] E. T. Akimova , R. Breen , D. M. Brazel , and M. C. Mills , “Gene‐Environment Dependencies Lead to Collider Bias in Models With Polygenic Scores,” Scientific Reports 11 (2021): 9457, 10.1038/s41598-021-89020-x. 33947934 PMC8097011

[28] C. Lockhart , J. Bright , Y. Ahmadzadeh , et al., “Twins Early Development Study (TEDS): A Genetically Sensitive Investigation of Mental Health Outcomes in the Mid‐Twenties,” JCPP Advances 3, no. 2 (2023): e12154, 10.1002/jcv2.12154. 37753150 PMC10519737

[29] J. Wardle , S. Carnell , C. M. Haworth , and R. Plomin , “Evidence for a Strong Genetic Influence on Childhood Adiposity Despite the Force of the Obesogenic Environment,” American Journal of Clinical Nutrition 87, no. 2 (2008): 398–404, 10.1093/ajcn/87.2.398. 18258631

[30] T. J. Cole , M. C. Bellizzi , K. M. Flegal , and W. H. Dietz , “Establishing a Standard Definition for Child Overweight and Obesity Worldwide: International Survey,” BMJ 320, no. 7244 (2000): 1240–1243, 10.1136/bmj.320.7244.1240. 10797032 PMC27365

[31] L. Yengo , J. Sidorenko , K. E. Kemper , et al., “Meta‐Analysis of Genome‐Wide Association Studies for Height and Body Mass Index in ∼700000 Individuals of European Ancestry,” Human Molecular Genetics 27, no. 20 (2018): 3641–3649, 10.1093/hmg/ddy271. 30124842 PMC6488973

[32] K. Lange , J. A. Kerr , T. Mansell , et al., “Can Adult Polygenic Scores Improve Prediction of Body Mass Index in Childhood?,” International Journal of Obesity (Lond) 46 (2022): 1375–1383, 10.1038/s41366-022-01130-2. 35505076

[33] F. Privé , J. Arbel , and B. J. Vilhjálmsson , “LDpred2: Better, Faster, Stronger,” Bioinformatics 36, no. 22–23 (2020): 5424–5431, 10.1093/bioinformatics/btaa1029. PMC8016455 33326037

[34] L. L. Birch , J. O. Fisher , K. Grimm‐Thomas , C. N. Markey , R. Sawyer , and S. L. Johnson , “Confirmatory Factor Analysis of the Child Feeding Questionnaire: A Measure of Parental Attitudes, Beliefs and Practices About Child Feeding and Obesity Proneness,” Appetite 36, no. 3 (2001): 201–210, 10.1006/appe.2001.0398. 11358344

[35] A. C. Petersen , L. Crockett , M. Richards , and A. Boxer , “A Self‐Report Measure of Pubertal Status: Reliability, Validity, and Initial Norms,” Journal of Youth and Adolescence 17, no. 2 (1988): 117–133, 10.1007/BF01537962. 24277579

[36] T. Asparouhov and B. Muthén , “Auxiliary Variables in Mixture Modeling: Three‐Step Approaches Using Mplus,” Structural Equation Modeling: A Multidisciplinary Journal 21, no. 3 (2014): 329–341, 10.1080/10705511.2014.915181.

[37] S. Steinsbekk , D. Belsky , I. C. Guzey , J. Wardle , and L. Wichstrøm , “Polygenic Risk, Appetite Traits, and Weight Gain in Middle Childhood: A Longitudinal Study,” JAMA Pediatrics 170, no. 2 (2016): e154472, 10.1001/jamapediatrics.2015.4472. 26830872 PMC5914161

[38] K. E. Westerman , D. I. Chasman , W. J. Gauderman , and A. Durvasula , “Pathway‐Specific Polygenic Scores Substantially Increase the Discovery of Gene‐Adiposity Interactions Impacting Liver Biomarkers,” Human Genetics and Genomics Advances 7, no. 1 (2025): 100515, 10.1016/j.xhgg.2025.100515. 40944320 PMC12508838

[39] R. J. F. Loos , “Genetic Causes of Obesity: Mapping a Path Forward,” Trends in Molecular Medicine 31, no. 4 (2025): 319–325, 10.1016/j.molmed.2025.02.002. 40089418

[40] J. B. Pingault , A. G. Allegrini , T. Odigie , et al., “Research Review: How to Interpret Associations Between Polygenic Scores, Environmental Risks, and Phenotypes,” Journal of Child Psychology and Psychiatry 63, no. 10 (2022): 1125–1139, 10.1111/jcpp.13607. 35347715 PMC9790749

[41] J. D. Tubbs , R. M. Porsch , S. S. Cherny , and P. C. Sham , “The Genes We Inherit and Those We Don't: Maternal Genetic Nurture and Child BMI Trajectories,” Behavior Genetics 50, no. 5 (2020): 310–319, 10.1007/s10519-020-10008-w. 32681386

[42] L. Wright , G. Shireby , T. T. Morris , N. M. Davies , and D. Bann , “The Association Between Parental BMI and Offspring Adiposity: A Genetically Informed Analysis of Trios,” PLoS Genetics 21, no. 8 (2025): e1011775, 10.1371/journal.pgen.1011775. 40763113 PMC12324121

[43] T. M. Schnurr , C. S. Morgen , D. Borisevich , et al., “The Influence of Transmitted and Non‐Transmitted Parental BMI‐Associated Alleles on the Risk of Overweight in Childhood,” Scientific Reports 10, no. 1 (2020): 4806, 10.1038/s41598-020-61719-3. 32179833 PMC7075975

[44] M. Herle , A. Pickles , N. Micali , M. Abdulkadir , and B. L. D. Stavola , “Parental Feeding and Childhood Genetic Risk for Obesity: Exploring Hypothetical Interventions With Causal Inference Methods,” International Journal of Obesity (Lond) 46, no. 7 (2022): 1271–1279, 10.1038/s41366-022-01106-2. PMC9239906 35306528

[45] M. Rios‐Leyvraz , N. Ortega , and A. Chiolero , “Reliability of Self‐Reported Height and Weight in Children: A School‐Based Cross‐Sectional Study and a Review,” Nutrients 15, no. 1 (2022): 75, 10.3390/nu15010075. 36615731 PMC9824624

