# Supplementary material for: Interaction Between Genetic Risk and Parental Feeding Practices in the Prediction of Overweight Across Adolescence
Source: Obesity (Silver Spring). 2026 May 11;34(7):1468–76. doi: 10.1002/oby.70217 (PMC13306130; doi:10.1002/oby.70217)
Supplement: Supplementary file 1 — Figure S1: Association of the polygenic score for BMI with BMI in TEDS (N = 6973). Figure S2: Heat map of correlations. Table S1: Pairwise correlations between variables in the study. Table S2: Model fit indices for latent class growth analyses of overweight status. Growth is defined by intercepts and linear and quadratic slopes. Table S3: Classification probabilities for the three‐class model. Average latent class probabilities for most likely latent class membership (row) by latent class (column). Table S4: Growth parameters for the three‐class model. Table S5: Full results of multinomial logistic regression models predicting class membership from the polygenic score for BMI (Model 1), parental feeding practices (Models 2a–4a), and their interactions (Models 2b–4b) (corresponding to Table 2). Table S6: Negative validation check: Restriction. Table S7: Negative validation check: Pressure to eat. Table S8: Negative validation check: Monitoring. Table S9: Unadjusted G × E model: Polygenic score for BMI × Restriction. Table S10: Unadjusted G × E model: Polygenic score for BMI × Pressure to eat. Table S11: Unadjusted G × E model: Polygenic score for BMI × Monitoring. Table S12: Follow‐up analyses excluding participants with underweight: Main effects of the polygenic score for BMI (Model 1), parental feeding practices (Models 2a–4a), and polygenic score for BMI × feeding practices (Models 2b–4b) in the prediction of class membership. [file OBY-34-1468-s001.docx]

Online Supplementary Information for:

**Interaction between genetic risk and parental feeding practices in the prediction of overweight across adolescence**

*Obesity*

Marthe de Roo^1^, MSc, Tina Kretschmer^2^, PhD, Clare Llewellyn^3^, PhD, Bonamy R. Oliver^4^, PhD, Catharina A. Hartman^5^, PhD

**Affiliations:** ^1^Faculty of Behavioral and Social Sciences, Department of Pedagogy and Educational Sciences, University of Groningen, Groningen, the Netherlands; ^2^Department of Psychology, Friedrich-Alexander University Erlangen-Nuremberg, Erlangen, Germany; ^3^Department of Behavioural Science and Health, University College London, London, UK; ^4^Department of Psychology and Human Development, UCL Institute of Education, University College London, London, UK; ^5^Interdisciplinary Center Psychopathology and Emotion Regulation (ICPE), University of Groningen, University Medical Center Groningen, Groningen, the Netherlands

**Contact info:**Marthe de Roo, Department of Pedagogy and Educational Sciences, Grote Rozenstraat 38, 9712 TJ Groningen, The Netherlands, [marthe.de.roo@rug.nl].

**Table of contents**

***Polygenic score for BMI***

3. Figure S1: Association of the polygenic score for BMI with BMI in TEDS

***Descriptives***

4. Figure S2: Heatmap of correlations

5. Table S1: Pairwise correlations between variables in the study

***Latent class growth analyses***

6. Table S2: Model fit indices

7. Table S3: Classification probabilities for the three-class model

8. Table S4: Growth parameters for the three-class model

***Main analyses: Full results***

9. Table S5: Full results of multinomial logistic regression models predicting class membership

***Negative validation check models***

12. Table S6: Negative validation check: Restriction

13. Table S7: Negative validation check: Pressure to eat

14. Table S8: Negative validation check: Monitoring

***Unadjusted G×E models***

15. Table S9: Polygenic score for BMI × Restriction

16. Table S10: Polygenic score for BMI × Pressure to eat

17. Table S11: Polygenic score for BMI × Monitoring

***Follow-up analyses***

18. Table S12: Results of multinomial logistic regression models excluding participants with underweight

**Figure S1.** Association of the polygenic score for BMI with BMI in TEDS (N = 6,973).

*Note.* Bars reflect the additional explained variance ($\Delta R^{2}$) in BMI at each time point when the polygenic score for BMI was added to a baseline model that included sex, age, and the first ten principal components.

**Figure S2.** Heatmap of correlations.

*Note*. n = 6,973. Missing values may occur for specific comparisons due to pairwise deletion of cases with missing values. Correlations between two binary variables are Phi coefficients, those between a binary and a continuous variable are point-biserial correlations, and all other correlations are Pearson’s *r*.

^a^ 0 = female, 1 = male.

^b^ 0 = non-overweight, 1 = overweight or obesity.

**Table S1.** Pairwise correlations between variables in the study.

|  | 1. | 2. | 3. | 4. | 5. | 6. | 7. | 8. | 9. | 10. | 11. | 12. | 13. | 14. | 15. |
| --- | --- | --- | --- | --- | --- | --- | --- | --- | --- | --- | --- | --- | --- | --- | --- |
| 1. Sex *(0=female, 1=male)* | 1.00 |  |  |  |  |  |  |  |  |  |  |  |  |  |  |
| 2. Age *(age 10)* | -.01 | 1.00 |  |  |  |  |  |  |  |  |  |  |  |  |  |
| 3. Socioeconomic status | -.01 | -.09* | 1.00 |  |  |  |  |  |  |  |  |  |  |  |  |
| 4. Polygenic score for BMI | .01 | .01 | -.14* | 1.00 |  |  |  |  |  |  |  |  |  |  |  |
| 5. Overweight (age 10) *(0=no, 1=yes)* | -.07* | .12* | -.10* | .22* | 1.00 |  |  |  |  |  |  |  |  |  |  |
| 6. Overweight (age 12) *(0=no, 1=yes)* | -.04* | .07* | -.07* | .21* | .64* | 1.00 |  |  |  |  |  |  |  |  |  |
| 7. Overweight (age 14) *(0=no, 1=yes)* | -.02 | .00 | -.09* | .22* | .42* | .48* | 1.00 |  |  |  |  |  |  |  |  |
| 8. Overweight (age 16) *(0=no, 1=yes)* | -.05* | .02 | -.08* | .27* | .42* | .44* | .51* | 1.00 |  |  |  |  |  |  |  |
| 9. Overweight (age 18) *(0=no, 1=yes)* | -.07* | .02 | -.11* | .24* | .34* | .35* | .42* | NA | 1.00 |  |  |  |  |  |  |
| 10. Restriction | .03* | -.07* | -.02 | .10* | .16* | .15* | .12* | .15* | .16* | 1.00 |  |  |  |  |  |
| 11. Pressure to eat | .05* | -.06* | -.03* | -.08* | -.17* | -.15* | -.11* | -.14* | -.14* | .15* | 1.00 |  |  |  |  |
| 12. Monitoring | .03 | -.05* | .03 | .02 | .03 | .03 | .02 | .03 | .03 | .26* | .01 | 1.00 |  |  |  |
| 13. Parental BMI | -.02 | .01 | -.20* | .24* | .22* | .20* | .18* | .16* | .26* | .07* | -.03 | -.02 | 1.00 |  |  |
| 14. Harsh discipline | .09* | -.02 | -.09* | .01 | -.03 | -.03 | -.03 | -.04 | NA | .06* | .08* | -.02 | .05* | 1.00 |  |
| 15. Positive parental feelings | -.03 | -.02 | .01 | -.02 | .00 | .01 | .02 | .05 | NA | -.06* | -.07* | .02 | .01 | -.16* | 1.00 |
| 16. Puberty scale | -.29* | .27* | -.07* | .09* | .18* | .14* | .09* | .09* | .15* | .01 | -.09* | .00 | .07* | -.04 | -.02 |

*Note*. n = 6,973. Missing values may occur for specific comparisons due to pairwise deletion of cases with missing values. Correlations between two binary variables are Phi coefficients, those between a binary and a continuous variable are point-biserial correlations, and all other correlations are Pearson’s *r*.

* *p* < .05.

**Table S2.** Model fit indices for latent class growth analyses of overweight status. Growth is defined by intercepts and linear and quadratic slopes.

| Measure | 1 Class | 2 Class | 3 Class | 4 Class |
| --- | --- | --- | --- | --- |
| Loglikelihood | -5043.6 | -4240.0 | **-4221.2** | -4215.2 |
| AIC | 10093.2 | 8493.9 | **8464.4** | 8460.3 |
| BIC | 10113.1 | 8540.3 | **8537.3** | 8559.7 |
| SSA-BIC | 10103.5 | 8518.0 | **8502.3** | 8512.0 |
| Entropy | - | .84 | **.84** | .82 |
| LMR-LRT, *p* value | - | <.001 | **<.001** | .61 |
| BLRT, *p* value | - | <.001 | **<.001** | .01 |
| Smallest class size (%) | - | 12.8% | **3.3%** | 3.9% |

*Note.* n = 5,568. Selected model is indicated in boldface. AIC=Akaike Information Criterion, BIC=Bayesian Information Criterion, SSA-BIC=Sample Size Adjusted BIC, LMR=Lo-Mendell-Rubin Adjusted Likelihood Ratio Test, BLRT=Bootstrap Likelihood Ratio Test.

**Table S3.** Classification probabilities for the three-class model. Average latent class probabilities for most likely latent class membership (row) by latent class (column).

| Class | Class Label | 1 | 2 | 3 |
| --- | --- | --- | --- | --- |
| 1 | Persistent Overweight | **0.85** | 0.11 | 0.05 |
| 2 | Non-Overweight | 0.02 | **0.96** | 0.03 |
| 3 | Adolescent-Onset Overweight | 0.16 | 0.12 | **0.71** |

**Table S4.** Growth parameters for the three-class model.

| Class | Class Label | n (%)^a^ | Latent growth factor | Estimate^b^ | *SE* | *p*-value |
| --- | --- | --- | --- | --- | --- | --- |
| 1 | Persistent | 604 (10.8%) | Intercept | -5.66 | 0.87 | <.001 |
|  | overweight |  | Linear slope | -9.64 | 2.06 | <.001 |
|  |  |  | Quadratic slope | 8.19 | 1.94 | <.001 |
| 2 | Non-overweight | 4779 (85.6%) | Intercept | -0.63 | 1.12 | .58 |
|  |  |  | Linear slope | -3.53 | 1.05 | .001 |
|  |  |  | Quadratic slope | 5.31 | 1.16 | <.001 |
| 3 | Adolescent-onset | 182 (3.3%) | Intercept | 0.00 | 0.00 | 1 |
|  | Overweight |  | Linear slope | 13.09 | 7.31 | .07 |
|  |  |  | Quadratic slope | -9.94 | 7.28 | .17 |

*Note.* *SE* = standard error.

^a^ Based on posterior probabilities.

**Table S5.** Full results of multinomial logistic regression models predicting class membership from the polygenic score for BMI (Model 1), parental feeding practices (Models 2a-4a), and their interactions (Models 2b-4b) (corresponding to Table 2).

|  |  | Adolescent-onset overweight versus non-overweight (ref) | |  | Persistent overweight versus non-overweight (ref) | |  | Persistent overweight versus adolescent-onset overweight (ref) | |
| --- | --- | --- | --- | --- | --- | --- | --- | --- | --- |
| **Model** |  | OR (*SE*) | 95% CI |  | OR (*SE)* | 95% CI |  | OR (*SE*) | 95% CI |
| **1. Main effect** | Sex^a^ | 1.71 (1.12) | [0.48, 6.16] |  | 0.79 (0.10) | [0.61, 1.01] |  | 0.46 (0.30) | [0.13, 1.64] |
| **Polygenic score** | Age | 0.97 (0.39) | [0.45, 2.13] |  | 1.37 (0.12)* | [1.16, 1.62] |  | 1.41 (0.58) | [0.63, 3.14] |
| **for BMI** | Socioeconomic status | 1.39 (0.75) | [0.49, 3.98] |  | 0.90 (0.06) | [0.79, 1.03] |  | 0.65 (0.35) | [0.22, 1.88] |
|  | Parental BMI | 1.13 (0.06)* | [1.01, 1.26] |  | 1.13 (0.02)* | [1.10, 1.16] |  | 1.00 (0.05) | [0.90, 1.11] |
|  | Puberty scale | 2.79 (1.33)* | [1.10, 7.10] |  | 2.00 (0.23)* | [1.60, 2.50] |  | 0.72 (0.33) | [0.29, 1.77] |
|  | Polygenic score for BMI | 3.01 (1.82) | [0.93, 9.81] |  | 2.14 (0.15)* | [1.86, 2.46] |  | 0.71 (0.43) | [0.22, 2.32] |
| **2a. Main effect** | Sex^a^ | 1.77 (1.23) | [0.46, 6.87] |  | 0.75 (0.10)* | [0.58, 0.97] |  | 0.43 (0.29) | [0.11, 1.62] |
| **Restriction** | Age | 1.06 (0.43) | [0.48, 2.35] |  | 1.42 (0.12)* | [1.20, 1.68] |  | 1.35 (0.56) | [0.60, 3.04] |
|  | Socioeconomic status | 1.46 (0.97) | [0.40, 5.34] |  | 0.90 (0.06) | [0.79, 1.03] |  | 0.62 (0.41) | [0.17, 2.27] |
|  | Parental BMI | 1.13 (0.07)* | [1.01, 1.28] |  | 1.13 (0.02)* | [1.09, 1.16] |  | 0.99 (0.06) | [0.89, 1.11] |
|  | Puberty scale | 2.56 (1.32) | [0.93, 7.04] |  | 1.98 (0.23)* | [1.57, 2.49] |  | 0.78 (0.39) | [0.29, 2.06] |
|  | Polygenic score for BMI | 2.85 (1.58) | [0.97, 8.42] |  | 2.08 (0.15)* | [1.80, 2.41] |  | 0.73 (0.41) | [0.25, 2.17] |
|  | Restriction | 2.02 (1.25) | [0.60, 6.78] |  | 1.62 (0.13)* | [1.38, 1.90] |  | 0.80 (0.52) | [0.23, 2.85] |
| **2b. Polygenic score** | Sex^a^ | 2.05 (1.89) | [0.34, 12.53] |  | 0.75 (0.10)* | [0.58, 0.97] |  | 0.37 (0.34) | [0.06, 2.28] |
| **for BMI ×** | Age | 1.06 (0.44) | [0.48, 2.38] |  | 1.42 (0.12)* | [1.19, 1.68] |  | 1.33 (0.56) | [0.58, 3.06] |
| **Restriction** | Socioeconomic status | 1.36 (0.77) | [0.45, 4.14] |  | 0.92 (0.07) | [0.80, 1.06] |  | 0.68 (0.39) | [0.22, 2.07] |
|  | Parental BMI | 1.14 (0.10) | [0.97, 1.34] |  | 1.13 (0.02)* | [1.09, 1.16] |  | 0.99 (0.09) | [0.84, 1.17] |
|  | Puberty scale | 2.76 (1.55) | [0.92, 8.29] |  | 2.01 (0.24)* | [1.59, 2.53] |  | 0.73 (0.40) | [0.25, 2.14] |
|  | Polygenic score for BMI | 2.61 (1.66) | [0.75, 9.07] |  | 2.07 (0.16)* | [1.78, 2.40] |  | 0.79 (0.51) | [0.23, 2.78] |
|  | Restriction | 1.51 (1.42) | [0.24, 9.50] |  | 1.59 (0.15)* | [1.32, 1.93] |  | 1.06 (1.03) | [0.16, 7.14] |
|  | PGS*_BMI_* × Restriction | 1.30 (1.12) | [0.24, 7.06] |  | 1.04 (0.10) | [0.85, 1.27] |  | 0.80 (0.72) | [0.14, 4.64] |
|  | Restriction × Socioeconomic status | 1.45 (0.83) | [0.47, 4.47] |  | 0.92 (0.08) | [0.78, 1.08] |  | 0.64 (0.38) | [0.20, 2.06] |
|  | Restriction × Parental BMI | 1.00 (0.09) | [0.83, 1.20] |  | 1.00 (0.02) | [0.97, 1.03] |  | 1.00 (0.10) | [0.83, 1.20] |
| **3a. Main effect** | Sex^a^ | 1.74 (1.19) | [0.46, 6.67] |  | 0.79 (0.10) | [0.61, 1.02] |  | 0.45 (0.31) | [0.12, 1.72] |
| **Pressure to eat** | Age | 0.99 (0.43) | [0.42, 2.30] |  | 1.34 (0.11)* | [1.14, 1.58] |  | 1.36 (0.59) | [0.58, 3.18] |
|  | Socioeconomic status | 1.44 (0.74) | [0.53, 3.92] |  | 0.85 (0.06)* | [0.75, 0.97] |  | 0.59 (0.31) | [0.22, 1.62] |
|  | Parental BMI | 1.14 (0.06)* | [1.02, 1.27] |  | 1.13 (0.02)* | [1.10, 1.16] |  | 1.00 (0.05) | [0.90, 1.10] |
|  | Puberty scale | 3.12 (1.65)* | [1.11, 8.77] |  | 1.94 (0.23)* | [1.54, 2.44] |  | 0.62 (0.32) | [0.23, 1.70] |
|  | Polygenic score for BMI | 3.30 (2.07) | [0.97, 11.28] |  | 2.12 (0.16)* | [1.84, 2.45] |  | 0.64 (0.40) | [0.19, 2.19] |
|  | Pressure to eat | 0.97 (0.47) | [0.37, 2.49] |  | 0.49 (0.04)* | [0.42, 0.58] |  | 0.51 (0.24) | [0.20, 1.29] |
| **3b. Polygenic score** | Sex^a^ | 1.72 (1.53) | [0.30, 9.83] |  | 0.79 (0.10) | [0.61, 1.02] |  | 0.46 (0.40) | [0.08, 2.58] |
| **for BMI × Pressure** | Age | 1.03 (0.52) | [0.38, 2.76] |  | 1.34 (0.11)* | [1.13, 1.58] |  | 1.30 (0.66) | [0.48, 3.53] |
| **to eat** | Socioeconomic status | 1.65 (1.40) | [0.32, 8.67] |  | 0.82 (0.06)* | [0.70, 0.96] |  | 0.50 (0.42) | [0.10, 2.57] |
|  | Parental BMI | 1.14 (0.09) | [0.98, 1.32] |  | 1.13 (0.02)* | [1.09, 1.16] |  | 0.99 (0.07) | [0.86, 1.15] |
|  | Puberty scale | 3.28 (2.03) | [0.97, 11.06] |  | 1.94 (0.24)* | [1.52, 2.47] |  | 0.59 (0.36) | [0.18, 1.94] |
|  | Polygenic score for BMI | 4.12 (4.08) | [0.59, 28.67] |  | 2.00 (0.17)* | [1.69, 2.37] |  | 0.49 (0.49) | [0.07, 3.50] |
|  | Pressure to eat | 1.27 (1.38) | [0.15, 10.73] |  | 0.53 (0.05)* | [0.44, 0.64] |  | 0.42 (0.45) | [0.05, 3.53] |
|  | PGS*_BMI_* × Pressure to eat | 0.93 (0.58) | [0.27, 3.14] |  | 0.83 (0.08) | [0.68, 1.01] |  | 0.90 (0.57) | [0.26, 3.14] |
|  | Pressure to eat × Socioeconomic status | 0.70 (0.48) | [0.19, 2.67] |  | 0.90 (0.09) | [0.74, 1.08] |  | 1.27 (0.88) | [0.33, 4.90] |
|  | Pressure to eat × Parental BMI | 0.96 (0.06) | [0.84, 1.08] |  | 0.99 (0.02) | [0.95, 1.03] |  | 1.03 (0.07) | [0.91, 1.17] |
| **4a. Main effect** | Sex^a^ | 1.72 (1.12) | [0.48, 6.17] |  | 0.78 (0.10) | [0.61, 1.00] |  | 0.45 (0.30) | [0.13, 1.62] |
| **Monitoring** | Age | 0.95 (0.40) | [0.42, 2.15] |  | 1.38 (0.12)* | [1.17, 1.64] |  | 1.46 (0.63) | [0.63, 3.39] |
|  | Socioeconomic status | 1.40 (0.77) | [0.48, 4.11] |  | 0.89 (0.06) | [0.78, 1.02] |  | 0.64 (0.36) | [0.22, 1.90] |
|  | Parental BMI | 1.13 (0.07)* | [1.01, 1.27] |  | 1.13 (0.02)* | [1.10, 1.16] |  | 1.00 (0.06) | [0.89, 1.11] |
|  | Puberty scale | 2.79 (1.42)* | [1.03, 7.58] |  | 2.00 (0.23)* | [1.60, 2.50] |  | 0.72 (0.35) | [0.27, 1.89] |
|  | Polygenic score for BMI | 2.94 (1.81) | [0.88, 9.83] |  | 2.13 (0.15)* | [1.85, 2.45] |  | 0.72 (0.45) | [0.22, 2.44] |
|  | Monitoring | 0.94 (0.38) | [0.43, 2.06] |  | 1.17 (0.09)* | [1.00, 1.36] |  | 1.24 (0.51) | [0.55, 2.78] |
| **4b. Polygenic score** | Sex^a^ | 1.77 (1.28) | [0.43, 7.32] |  | 0.80 (0.10) | [0.62, 1.03] |  | 0.45 (0.33) | [0.11, 1.88] |
| **for BMI × Monitoring** | Age | 0.94 (0.47) | [0.35, 2.48] |  | 1.39 (0.12)* | [1.17, 1.64] |  | 1.48 (0.75) | [0.55, 3.98] |
|  | Socioeconomic status | 1.48 (0.90) | [0.45, 4.87] |  | 0.90 (0.06) | [0.78, 1.02] |  | 0.61 (0.37) | [0.18, 2.02] |
|  | Parental BMI | 1.12 (0.08) | [0.98, 1.28] |  | 1.13 (0.02)* | [1.10, 1.16] |  | 1.01 (0.07) | [0.89, 1.15] |
|  | Puberty scale | 3.05 (1.81) | [0.95, 9.77] |  | 2.01 (0.23)* | [1.60, 2.53] |  | 0.66 (0.38) | [0.21, 2.06] |
|  | Polygenic score for BMI | 3.19 (2.33) | [0.76, 13.33] |  | 2.10 (0.15)* | [1.82, 2.41] |  | 0.66 (0.48) | [0.16, 2.75] |
|  | Monitoring | 1.16 (0.92) | [0.24, 5.53] |  | 1.11 (0.10) | [0.94, 1.32] |  | 0.96 (0.78) | [0.19, 4.75] |
|  | PGS*_BMI_* × Monitoring | 0.90 (0.55) | [0.27, 2.97] |  | 1.17 (0.10) | [0.99, 1.40] |  | 1.31 (0.81) | [0.39, 4.43] |
|  | Monitoring × Socioeconomic status | 0.77 (0.42) | [0.26, 2.23] |  | 0.93 (0.08) | [0.79, 1.10] |  | 1.22 (0.66) | [0.42, 3.53] |
|  | Monitoring × Parental BMI | 1.07 (0.11) | [0.87, 1.30] |  | 0.99 (0.02) | [0.96, 1.02] |  | 0.93 (0.10) | [0.76, 1.14] |

*Note.* OR = odds ratio, *SE* = standard error, CI = confidence interval, PGS*_BMI_* = polygenic score for BMI. The first-named class served as the reference category (ref). Analyses were based on n = 5,568 participants. All models including the polygenic score for BMI were adjusted for the first 10 principal components and chip type.
^a^ 0 = female, 1 = male.
* indicates *p* < .05.

**Table S6.** Negative validation check: Restriction.

|  | Adolescent-onset overweight versus non-overweight (ref) | |  | Persistent overweight versus non-overweight (ref) | |  | Persistent overweight versus adolescent-onset overweight (ref) | |
| --- | --- | --- | --- | --- | --- | --- | --- | --- |
|  | OR (*SE*) | OR 95% CI |  | OR (*SE*) | OR 95% CI |  | OR (*SE*) | OR 95% CI |
| Sex *(reference = female)* | 1.72 (1.18) | [0.45, 6.60] |  | 0.77 (0.10)* | [0.60, 0.99] |  | 0.45 (0.31) | [0.12, 1.70] |
| Age | 1.02 (0.43) | [0.45, 2.32] |  | 1.42 (0.12)* | [1.20, 1.68] |  | 1.39 (0.60) | [0.60, 3.25] |
| Socioeconomic status | 1.38 (0.71) | [0.50, 3.80] |  | 0.89 (0.06) | [0.77, 1.02] |  | 0.64 (0.34) | [0.23, 1.79] |
| Parental BMI | 1.14 (0.08)* | [1.00, 1.30] |  | 1.13 (0.02)* | [1.10, 1.16] |  | 0.99 (0.07) | [0.87, 1.13] |
| Puberty scale | 2.45 (1.40) | [0.79, 7.53] |  | 2.01 (0.24)* | [1.59, 2.53] |  | 0.82 (0.46) | [0.27, 2.46] |
| Polygenic score for BMI | 2.60 (1.16)* | [1.08, 6.24] |  | 2.10 (0.15)* | [1.82, 2.41] |  | 0.81 (0.36) | [0.34, 1.93] |
| **Restriction** | **1.80 (1.18)** | **[0.50, 6.47]** |  | **1.64 (0.13)*** | **[1.40, 1.92]** |  | **0.91 (0.62)** | **[0.24, 3.45]** |
| Negative parental discipline | 1.34 (0.66) | [0.51, 3.52] |  | 0.81 (0.09) | [0.66, 1.00] |  | 0.61 (0.31) | [0.22, 1.64] |
| Positive parental feelings | 0.70 (0.28) | [0.32, 1.51] |  | 1.09 (0.13) | [0.86, 1.37] |  | 1.55 (0.62) | [0.71, 3.41] |

*Note.* Analyses were based on n = 5,568 participants. ORs reflect the likelihood of belonging to each higher-risk trajectory relative to the reference class (ref). All models including the polygenic score for BMI were adjusted for the first 10 principal components of the genetic data and chip type.

* indicates *p* < .05.

**Table S7.** Negative validation check: Pressure to eat.

|  | Adolescent-onset overweight versus non-overweight (ref) | |  | Persistent overweight versus non-overweight (ref) | |  | Persistent overweight versus adolescent-onset overweight (ref) | |
| --- | --- | --- | --- | --- | --- | --- | --- | --- |
|  | OR (*SE*) | OR 95% CI |  | OR (*SE*) | OR 95% CI |  | OR (*SE*) | OR 95% CI |
| Sex *(reference = female)* | 1.75 (1.21) | [0.45, 6.80] |  | 0.81 (0.11) | [0.63, 1.04] |  | 0.46 (0.32) | [0.12, 1.78] |
| Age | 0.9 (0.42) | [0.36, 2.25] |  | 1.34 (0.11)* | [1.13, 1.58] |  | 1.49 (0.71) | [0.58, 3.81] |
| Socioeconomic status | 1.29 (0.65) | [0.48, 3.48] |  | 0.84 (0.06)* | [0.74, 0.97] |  | 0.65 (0.33) | [0.24, 1.77] |
| Parental BMI | 1.13 (0.07)* | [1.00, 1.28] |  | 1.13 (0.02)* | [1.10, 1.16] |  | 1.00 (0.06) | [0.89, 1.13] |
| Puberty scale | 3.07 (1.64)* | [1.07, 8.75] |  | 1.95 (0.23)* | [1.54, 2.47] |  | 0.64 (0.33) | [0.23, 1.76] |
| Polygenic score for BMI | 2.78 (1.64) | [0.87, 8.83] |  | 2.14 (0.16)* | [1.85, 2.47] |  | 0.77 (0.45) | [0.24, 2.43] |
| **Pressure to eat** | **0.86 (0.40)** | **[0.34, 2.15]** |  | **0.50 (0.04)*** | **[0.42, 0.59]** |  | **0.58 (0.27)** | **[0.23, 1.45]** |
| Negative parental discipline | 1.28 (0.59) | [0.52, 3.14] |  | 0.86 (0.09) | [0.70, 1.05] |  | 0.67 (0.32) | [0.26, 1.70] |
| Positive parental feelings | 0.66 (0.31) | [0.26, 1.65] |  | 1.02 (0.12) | [0.82, 1.28] |  | 1.56 (0.74) | [0.61, 3.97] |

*Note.* Analyses were based on n = 5,568 participants. ORs reflect the likelihood of belonging to each higher-risk trajectory relative to the reference class (ref). All models including the polygenic score for BMI were adjusted for the first 10 principal components of the genetic data and chip type.

* indicates *p* < .05.

**Table S8.** Negative validation check: Monitoring.

|  | Adolescent-onset overweight versus non-overweight (ref) | |  | Persistent overweight versus non-overweight (ref) | |  | Persistent overweight versus adolescent-onset overweight (ref) | |
| --- | --- | --- | --- | --- | --- | --- | --- | --- |
|  | OR (*SE*) | OR 95% CI |  | OR (*SE*) | OR 95% CI |  | OR (*SE*) | OR 95% CI |
| Sex *(reference = female)* | 1.73 (1.23) | [0.43, 7.00] |  | 0.80 (0.10) | [0.62, 1.03] |  | 0.46 (0.33) | [0.12, 1.87] |
| Age | 0.87 (0.42) | [0.34, 2.22] |  | 1.38 (0.12)* | [1.17, 1.64] |  | 1.60 (0.79) | [0.60, 4.23] |
| Socioeconomic status | 1.31 (0.69) | [0.47, 3.65] |  | 0.88 (0.06) | [0.77, 1.01] |  | 0.68 (0.36) | [0.24, 1.93] |
| Parental BMI | 1.13 (0.07)* | [1.00, 1.28] |  | 1.13 (0.02)* | [1.10, 1.16] |  | 1.00 (0.06) | [0.88, 1.13] |
| Puberty scale | 2.77 (1.65) | [0.86, 8.91] |  | 2.02 (0.23)* | [1.61, 2.53] |  | 0.73 (0.43) | [0.23, 2.29] |
| Polygenic score for BMI | 2.60 (1.38) | [0.92, 7.36] |  | 2.14 (0.15)* | [1.87, 2.46] |  | 0.83 (0.44) | [0.29, 2.34] |
| **Monitoring** | **0.95 (0.41)** | **[0.40, 2.23]** |  | **1.17 (0.09)** | **[1.00, 1.36]** |  | **1.23 (0.55)** | **[0.51, 2.97]** |
| Negative parental discipline | 1.33 (0.59) | [0.56, 3.18] |  | 0.83 (0.09) | [0.68, 1.01] |  | 0.62 (0.29) | [0.25, 1.54] |
| Positive parental feelings | 0.65 (0.29) | [0.27, 1.57] |  | 1.07 (0.13) | [0.85, 1.35] |  | 1.64 (0.75) | [0.67, 4.03] |

*Note.* Analyses were based on n = 5,568 participants. ORs reflect the likelihood of belonging to each higher-risk trajectory relative to the reference class (ref). All models including the polygenic score for BMI were adjusted for the first 10 principal components of the genetic data and chip type.

* indicates *p* < .05.

**Table S9.** Unadjusted G×E model: Polygenic score for BMI × Restriction.

|  | Adolescent-onset overweight versus non-overweight (ref) | |  | Persistent overweight versus non-overweight (ref) | |  | Persistent overweight versus adolescent-onset overweight (ref) | |
| --- | --- | --- | --- | --- | --- | --- | --- | --- |
|  | OR (*SE*) | OR 95% CI |  | OR (*SE*) | OR 95% CI |  | OR (*SE*) | OR 95% CI |
| Sex *(reference = female)* | 1.76 (1.30) | [0.41, 7.45] |  | 0.76 (0.10)* | [0.59, 0.97] |  | 0.43 (0.32) | [0.10, 1.80] |
| Age | 1.04 (0.42) | [0.47, 2.30] |  | 1.42 (0.12)* | [1.21, 1.68] |  | 1.38 (0.57) | [0.61, 3.09] |
| Socioeconomic status | 1.43 (0.77) | [0.49, 4.12] |  | 0.90 (0.06) | [0.79, 1.03] |  | 0.63 (0.34) | [0.22, 1.84] |
| Parental BMI | 1.13 (0.07)* | [1.00, 1.28] |  | 1.13 (0.02)* | [1.10, 1.16] |  | 1.00 (0.06) | [0.89, 1.12] |
| Puberty scale | 2.59 (1.47) | [0.85, 7.90] |  | 1.99 (0.24)* | [1.58, 2.51] |  | 0.77 (0.42) | [0.26, 2.27] |
| Polygenic score for BMI | 3.09 (1.84) | [0.96, 9.95] |  | 2.05 (0.16)* | [1.77, 2.38] |  | 0.67 (0.40) | [0.20, 2.18] |
| Restriction | 2.26 (1.83) | [0.46, 11.06] |  | 1.58 (0.14)* | [1.32, 1.89] |  | 0.70 (0.59) | [0.13, 3.68] |
| **Polygenic score for BMI × Restriction** | **0.92 (0.51)** | **[0.31, 2.71]** |  | **1.06 (0.10)** | **[0.89, 1.28]** |  | **1.15 (0.65)** | **[0.38, 3.50]** |

*Note.* Analyses were based on n = 5,568 participants. ORs reflect the likelihood of belonging to each higher-risk trajectory relative to the reference class (ref). All models including the polygenic score for BMI were adjusted for the first 10 principal components of the genetic data and chip type.

* indicates *p* < .05.

**Table S10.** Unadjusted G×E model: Polygenic score for BMI × Pressure to eat.

|  | Adolescent-onset overweight versus non-overweight (ref) | |  | Persistent overweight versus non-overweight (ref) | |  | Persistent overweight versus adolescent-onset overweight (ref) | |
| --- | --- | --- | --- | --- | --- | --- | --- | --- |
|  | OR (*SE*) | OR 95% CI |  | OR (*SE*) | OR 95% CI |  | OR (*SE*) | OR 95% CI |
| Sex *(reference = female)* | 1.88 (1.33) | [0.47, 7.51] |  | 0.79 (0.10) | [0.61, 1.02] |  | 0.42 (0.29) | [0.11, 1.65] |
| Age | 1.01 (0.42) | [0.44, 2.29] |  | 1.34 (0.11)* | [1.13, 1.58] |  | 1.33 (0.56) | [0.58, 3.04] |
| Socioeconomic status | 1.44 (0.77) | [0.51, 4.09] |  | 0.85 (0.06)* | [0.74, 0.97] |  | 0.59 (0.31) | [0.21, 1.68] |
| Parental BMI | 1.14 (0.06)* | [1.02, 1.27] |  | 1.13 (0.02)* | [1.10, 1.17] |  | 1.00 (0.05) | [0.90, 1.11] |
| Puberty scale | 3.18 (1.74)* | [1.09, 9.27] |  | 1.95 (0.23)* | [1.54, 2.46] |  | 0.61 (0.33) | [0.22, 1.74] |
| Polygenic score for BMI | 3.57 (2.46) | [0.92, 13.79] |  | 2.01 (0.16)* | [1.72, 2.35] |  | 0.56 (0.39) | [0.15, 2.17] |
| Pressure to eat | 0.99 (0.66) | [0.27, 3.68] |  | 0.53 (0.05)* | [0.44, 0.63] |  | 0.53 (0.36) | [0.14, 1.98] |
| **Polygenic score for BMI × Pressure to eat** | **0.88 (0.45)** | **[0.32, 2.40]** |  | **0.83 (0.08)** | **[0.69, 1.01]** |  | **0.95 (0.49)** | **[0.35, 2.61]** |

*Note.* Analyses were based on n = 5,568 participants. ORs reflect the likelihood of belonging to each higher-risk trajectory relative to the reference class (ref). All models including the polygenic score for BMI were adjusted for the first 10 principal components of the genetic data and chip type.

* indicates *p* < .05.

**Table S11.** Unadjusted G×E model: Polygenic score for BMI × Monitoring.

|  | Adolescent-onset overweight versus non-overweight (ref) | |  | Persistent overweight versus non-overweight (ref) | |  | Persistent overweight versus adolescent-onset overweight (ref) | |
| --- | --- | --- | --- | --- | --- | --- | --- | --- |
|  | OR (*SE*) | OR 95% CI |  | OR (*SE*) | OR 95% CI |  | OR (*SE*) | OR 95% CI |
| Sex *(reference = female)* | 1.75 (1.16) | [0.47, 6.44] |  | 0.79 (0.10) | [0.61, 1.01] |  | 0.45 (0.30) | [0.12, 1.66] |
| Age | 0.95 (0.39) | [0.43, 2.11] |  | 1.39 (0.12)* | [1.18, 1.64] |  | 1.47 (0.62) | [0.64, 3.34] |
| Socioeconomic status | 1.42 (0.76) | [0.50, 4.05] |  | 0.89 (0.06) | [0.78, 1.02] |  | 0.63 (0.34) | [0.22, 1.81] |
| Parental BMI | 1.14 (0.07)* | [1.01, 1.27] |  | 1.13 (0.02)* | [1.10, 1.16] |  | 1.00 (0.06) | [0.89, 1.11] |
| Puberty scale | 2.80 (1.40)* | [1.05, 7.44] |  | 2.01 (0.23)* | [1.60, 2.51] |  | 0.72 (0.35) | [0.28, 1.86] |
| Polygenic score for BMI | 2.93 (1.85) | [0.85, 10.09] |  | 2.10 (0.15)* | [1.83, 2.42] |  | 0.72 (0.46) | [0.21, 2.50] |
| Monitoring | 0.92 (0.46) | [0.34, 2.47] |  | 1.10 (0.09) | [0.93, 1.30] |  | 1.20 (0.63) | [0.43, 3.37] |
| **Polygenic score for BMI × Monitoring** | **1.19 (0.56)** | **[0.48, 2.98]** |  | **1.17 (0.10)** | **[0.99, 1.39]** |  | **0.98 (0.46)** | **[0.39, 2.46]** |

*Note.* Analyses were based on n = 5,568 participants. ORs reflect the likelihood of belonging to each higher-risk trajectory relative to the reference class (ref). All models including the polygenic score for BMI were adjusted for the first 10 principal components of the genetic data and chip type.

* indicates *p* < .05.

**Table S12.** Follow-up analyses excluding participants with underweight: Main effects of the polygenic score for BMI (Model 1), parental feeding practices (Models 2a-4a), and polygenic score for BMI × feeding practices (Models 2b-4b) in the prediction of class membership.

|  |  | Adolescent-onset overweight versus non-overweight (ref) | |  | Persistent overweight versus non-overweight (ref) | |  | Persistent overweight versus adolescent-onset overweight (ref) | |
| --- | --- | --- | --- | --- | --- | --- | --- | --- | --- |
| **Model** |  | OR (*SE*) | 95% CI |  | OR (*SE)* | 95% CI |  | OR (*SE*) | 95% CI |
| **1. Main effect** | Sex^a^ | 1.56 (1.04) | [0.42, 5.77] |  | 0.77 (0.10)* | [0.60, 0.99] |  | 0.50 (0.33) | [0.14, 1.81] |
| **Polygenic score** | Age | 0.99 (0.42) | [0.43, 2.25] |  | 1.38 (0.13)* | [1.15, 1.65] |  | 1.40 (0.60) | [0.60, 3.25] |
| **for BMI** | Socioeconomic status | 1.34 (0.76) | [0.45, 4.04] |  | 0.90 (0.06) | [0.79, 1.03] |  | 0.67 (0.38) | [0.22, 2.04] |
|  | Parental BMI | 1.12 (0.06)* | [1.01, 1.24] |  | 1.13 (0.02)* | [1.10, 1.16] |  | 1.01 (0.05) | [0.91, 1.11] |
|  | Puberty scale | 2.94 (1.53)* | [1.06, 8.17] |  | 1.96 (0.23)* | [1.55, 2.47] |  | 0.67 (0.33) | [0.25, 1.75] |
|  | Polygenic score for BMI | 3.23 (2.07) | [0.92, 11.37] |  | 2.14 (0.15)* | [1.86, 2.47] |  | 0.66 (0.43) | [0.19, 2.34] |
| **2a. Main effect** | Sex^a^ | 1.64 (1.12) | [0.43, 6.23] |  | 0.74 (0.10)* | [0.58, 0.96] |  | 0.46 (0.31) | [0.12, 1.70] |
| **Restriction** | Age | 1.08 (0.45) | [0.48, 2.44] |  | 1.43 (0.14)* | [1.19, 1.73] |  | 1.33 (0.56) | [0.58, 3.03] |
|  | Socioeconomic status | 1.43 (0.93) | [0.40, 5.13] |  | 0.90 (0.06) | [0.79, 1.03] |  | 0.63 (0.41) | [0.18, 2.27] |
|  | Parental BMI | 1.12 (0.07) | [0.99, 1.27] |  | 1.12 (0.02)* | [1.09, 1.16] |  | 1.00 (0.06) | [0.89, 1.12] |
|  | Puberty scale | 2.68 (1.48) | [0.91, 7.89] |  | 1.94 (0.24)* | [1.53, 2.47] |  | 0.73 (0.38) | [0.26, 2.01] |
|  | Polygenic score for BMI | 3.09 (1.87) | [0.94, 10.14] |  | 2.09 (0.15)* | [1.81, 2.41] |  | 0.68 (0.41) | [0.21, 2.21] |
|  | Restriction | 1.98 (1.15) | [0.64, 6.16] |  | 1.64 (0.13)* | [1.41, 1.91] |  | 0.83 (0.50) | [0.25, 2.69] |
| **2b. Polygenic score** | Sex^a^ | 1.77 (1.39) | [0.38, 8.28] |  | 0.74 (0.10)* | [0.58, 0.96] |  | 0.42 (0.33) | [0.09, 1.98] |
| **for BMI ×** | Age | 1.06 (0.43) | [0.48, 2.36] |  | 1.43 (0.14)* | [1.18, 1.73] |  | 1.35 (0.57) | [0.59, 3.08] |
| **Restriction** | Socioeconomic status | 1.23 (0.74) | [0.38, 3.97] |  | 0.93 (0.07) | [0.80, 1.08] |  | 0.75 (0.46) | [0.23, 2.46] |
|  | Parental BMI | 1.13 (0.10) | [0.95, 1.33] |  | 1.12 (0.02)* | [1.09, 1.16] |  | 1.00 (0.09) | [0.84, 1.18] |
|  | Puberty scale | 2.73 (1.52) | [0.92, 8.13] |  | 1.96 (0.25)* | [1.53, 2.51] |  | 0.72 (0.39) | [0.25, 2.06] |
|  | Polygenic score for BMI | 2.93 (1.90) | [0.82, 10.43] |  | 2.08 (0.16)* | [1.79, 2.42] |  | 0.71 (0.47) | [0.20, 2.56] |
|  | Restriction | 1.72 (1.75) | [0.24, 12.60] |  | 1.62 (0.16)* | [1.33, 1.98] |  | 0.94 (0.99) | [0.12, 7.47] |
|  | PGS*_BMI_* × Restriction | 1.13 (0.87) | [0.25, 5.10] |  | 1.03 (0.10) | [0.85, 1.25] |  | 0.92 (0.74) | [0.19, 4.49] |
|  | Restriction × Socioeconomic status | 1.42 (0.87) | [0.43, 4.72] |  | 0.91 (0.09) | [0.75, 1.10] |  | 0.64 (0.42) | [0.18, 2.30] |
|  | Restriction × Parental BMI | 1.00 (0.09) | [0.84, 1.19] |  | 1.00 (0.02) | [0.97, 1.03] |  | 1.00 (0.09) | [0.84, 1.19] |
| **3a. Main effect** | Sex^a^ | 1.55 (1.08) | [0.39, 6.10] |  | 0.78 (0.10) | [0.61, 1.00] |  | 0.50 (0.35) | [0.13, 1.97] |
| **Pressure to eat** | Age | 1.01 (0.45) | [0.42, 2.42] |  | 1.34 (0.13)* | [1.11, 1.62] |  | 1.34 (0.60) | [0.55, 3.23] |
|  | Socioeconomic status | 1.43 (0.76) | [0.51, 4.04] |  | 0.85 (0.06)* | [0.75, 0.97] |  | 0.60 (0.31) | [0.21, 1.68] |
|  | Parental BMI | 1.12 (0.06)* | [1.01, 1.25] |  | 1.13 (0.02)* | [1.10, 1.16] |  | 1.00 (0.05) | [0.91, 1.11] |
|  | Puberty scale | 3.31 (1.90)* | [1.08, 10.18] |  | 1.89 (0.23)* | [1.49, 2.39] |  | 0.57 (0.32) | [0.19, 1.68] |
|  | Polygenic score for BMI | 3.74 (2.45)* | [1.04, 13.49] |  | 2.12 (0.16)* | [1.84, 2.45] |  | 0.57 (0.37) | [0.16, 2.03] |
|  | Pressure to eat | 1.04 (0.53) | [0.38, 2.82] |  | 0.49 (0.04)* | [0.41, 0.58] |  | 0.47 (0.24) | [0.18, 1.26] |
| **3b. Polygenic score** | Sex^a^ | 1.51 (1.24) | [0.30, 7.52] |  | 0.78 (0.10) | [0.60, 1.00] |  | 0.51 (0.42) | [0.10, 2.55] |
| **for BMI × Pressure** | Age | 1.03 (0.46) | [0.43, 2.45] |  | 1.34 (0.13)* | [1.11, 1.62] |  | 1.30 (0.58) | [0.55, 3.11] |
| **to eat** | Socioeconomic status | 1.63 (0.95) | [0.52, 5.12] |  | 0.82 (0.07)* | [0.70, 0.96] |  | 0.50 (0.29) | [0.16, 1.57] |
|  | Parental BMI | 1.12 (0.06)* | [1.00, 1.25] |  | 1.13 (0.02)* | [1.09, 1.16] |  | 1.00 (0.06) | [0.90, 1.12] |
|  | Puberty scale | 3.45 (2.22) | [0.98, 12.17] |  | 1.89 (0.23)* | [1.49, 2.39] |  | 0.55 (0.34) | [0.16, 1.86] |
|  | Polygenic score for BMI | 4.64 (3.11)* | [1.25, 17.22] |  | 2.01 (0.16)* | [1.72, 2.36] |  | 0.43 (0.29) | [0.12, 1.63] |
|  | Pressure to eat | 1.38 (0.90) | [0.38, 4.98] |  | 0.52 (0.05)* | [0.43, 0.63] |  | 0.38 (0.25) | [0.10, 1.36] |
|  | PGS*_BMI_* × Pressure to eat | 0.91 (0.40) | [0.38, 2.15] |  | 0.85 (0.09) | [0.69, 1.05] |  | 0.94 (0.43) | [0.38, 2.32] |
|  | Pressure to eat × Socioeconomic status | 0.69 (0.28) | [0.31, 1.55] |  | 0.89 (0.09) | [0.72, 1.09] |  | 1.28 (0.54) | [0.56, 2.93] |
|  | Pressure to eat × Parental BMI | 0.96 (0.06) | [0.85, 1.08] |  | 0.99 (0.02) | [0.96, 1.02] |  | 1.03 (0.07) | [0.91, 1.17] |
| **4a. Main effect** | Sex^a^ | 1.57 (1.04) | [0.43, 5.77] |  | 0.77 (0.10)* | [0.60, 0.99] |  | 0.49 (0.32) | [0.14, 1.79] |
| **Monitoring** | Age | 0.96 (0.43) | [0.40, 2.31] |  | 1.39 (0.13)* | [1.16, 1.67] |  | 1.44 (0.66) | [0.59, 3.55] |
|  | Socioeconomic status | 1.34 (0.82) | [0.41, 4.45] |  | 0.90 (0.06) | [0.78, 1.02] |  | 0.67 (0.41) | [0.20, 2.22] |
|  | Parental BMI | 1.12 (0.06)* | [1.00, 1.26] |  | 1.13 (0.02)* | [1.10, 1.16] |  | 1.00 (0.06) | [0.90, 1.12] |
|  | Puberty scale | 2.94 (1.60)* | [1.01, 8.55] |  | 1.96 (0.24)* | [1.55, 2.48] |  | 0.67 (0.35) | [0.24, 1.84] |
|  | Polygenic score for BMI | 3.15 (2.22) | [0.79, 12.51] |  | 2.14 (0.15)* | [1.86, 2.46] |  | 0.68 (0.48) | [0.17, 2.71] |
|  | Monitoring | 0.96 (0.41) | [0.42, 2.20] |  | 1.16 (0.09)* | [1.00, 1.35] |  | 1.20 (0.51) | [0.52, 2.78] |
| **4b. Polygenic score** | Sex^a^ | 1.54 (1.13) | [0.36, 6.52] |  | 0.79 (0.10) | [0.61, 1.01] |  | 0.51 (0.38) | [0.12, 2.18] |
| **for BMI × Monitoring** | Age | 0.95 (0.52) | [0.32, 2.80] |  | 1.39 (0.13)* | [1.16, 1.67] |  | 1.47 (0.82) | [0.49, 4.39] |
|  | Socioeconomic status | 1.42 (0.90) | [0.41, 4.94] |  | 0.89 (0.06) | [0.78, 1.02] |  | 0.63 (0.40) | [0.18, 2.21] |
|  | Parental BMI | 1.10 (0.07) | [0.98, 1.25] |  | 1.13 (0.02)* | [1.10, 1.16] |  | 1.02 (0.06) | [0.91, 1.15] |
|  | Puberty scale | 3.11 (2.03) | [0.87, 11.16] |  | 1.97 (0.24)* | [1.56, 2.49] |  | 0.63 (0.40) | [0.19, 2.17] |
|  | Polygenic score for BMI | 3.52 (2.57) | [0.84, 14.75] |  | 2.10 (0.15)* | [1.83, 2.42] |  | 0.60 (0.44) | [0.14, 2.51] |
|  | Monitoring | 1.26 (1.01) | [0.26, 6.05] |  | 1.10 (0.10) | [0.92, 1.31] |  | 0.87 (0.72) | [0.18, 4.35] |
|  | PGS*_BMI_* × Monitoring | 0.84 (0.49) | [0.27, 2.61] |  | 1.18 (0.11) | [0.98, 1.42] |  | 1.41 (0.83) | [0.45, 4.45] |
|  | Monitoring × Socioeconomic status | 0.76 (0.38) | [0.29, 2.04] |  | 0.93 (0.08) | [0.79, 1.10] |  | 1.22 (0.61) | [0.46, 3.27] |
|  | Monitoring × Parental BMI | 1.07 (0.11) | [0.88, 1.32] |  | 0.99 (0.02) | [0.96, 1.02] |  | 0.92 (0.10) | [0.75, 1.13] |

*Note.* OR = odds ratio, *SE* = standard error, CI = confidence interval, PGS*_BMI_* = polygenic score for BMI. ORs reflect the likelihood of belonging to each higher-risk trajectory relative to the reference class (ref). Participants with underweight (based on IOTF grade 2 thinness) at >=2 assessments were excluded (n = 68). Analyses were based on n = 5,500 participants. All models including the polygenic score for BMI were adjusted for the first 10 principal components of the genetic data and chip type.
^a^ 0 = female, 1 = male.
* indicates *p* < .05.
